# Trends and Causes of Raw Water Quality Indicators in the Five Most Famous Lakes of Jiangsu Province, China

**DOI:** 10.3390/ijerph19031580

**Published:** 2022-01-29

**Authors:** Yajun Chang, Zheyuan Feng, Jixiang Liu, Junfang Sun, Linhe Sun, Qiang Tang, Dongrui Yao

**Affiliations:** 1 Jiangsu Key Laboratory for the Research and Utilization of Plant Resources, Institute of Botany, Jiangsu Province and Chinese Academy of Sciences (Nanjing Botanical Garden Mem. Sun Yat-Sen), Nanjing 210014, China; changyj@cnbg.net (Y.C.); ljx891654338@163.com (J.L.); linhesun@cnbg.net (L.S.); 2 Jiangsu Engineering Research Center of Aquatic Plant Resources and Water Environment Remediation, Nanjing 210014, China; 3 School of Rail Transportation, Soochow University, Suzhou 215131, China; fengzheyuan1999@sina.com (Z.F.); tangqiang@suda.edu.cn (Q.T.); 4 Dongwu Business School, Soochow University, Suzhou 215021, China

**Keywords:** water quality, water pollution, phytoplankton density, zooplankton density, comprehensive pollution index

## Abstract

Due to pollutants from industrial and agricultural activities, the lakes in China are faced with ecological and environmental problems. The five most famous lakes of Jiangsu Province, Taihu Lake, Gehu Lake, Gaobaoshaobo Lake, Hongze Lake, and Luoma Lake, have long-term fixed monitoring points for water body-related indicators. Over a five-year period, the monitoring showed that Gehu Lake had the highest average total nitrogen (TN) and total phosphorus (TP) concentrations among all lakes which were close to the Grade V critical value of the China’s Environmental Quality Standards for Surface Water (CEQSW). The NH_3_-N concentrations in all lakes were Grade IV according to the China’s Water Quality Standard for Drinking Water Sources (CWQSDWS) and Grade II according to the CEQSW. In addition, although TP concentrations in Taihu Lake did not exceed Grade V in the CEQSW, TP removal was the main factor controlling eutrophication. It was also found that the petroleum concentrations in all lakes were lower than the Grade I according to the CEQSW. Despite this relatively low petroleum pollution, the concentration of petroleum was negatively correlated with the phytoplankton densities in all lakes. This indicated that phytoplankton density was very sensitive to petroleum concentration. For heavy metals, the concentrations of Pb, Cu, As, and Cd in all lakes were significantly lower than Grade I (CEQSW) from 2013 to 2017. However, the accumulated heavy metals in sediments will remain an important pollution source affecting water quality and aquatic products in the future. The comprehensive pollution index analysis showed that the five lakes were often moderately polluted, indicating that the protection of lake resources in China should not be relaxed for a long time in the future.

## 1. Introduction

Lakes serve various functions that are essential to human survival and economic development, such as regulating runoff and flooding, water storage and irrigation, water supply, fishery production, shipping of goods, climate regulation, and maintenance of regional ecosystems [1]. However, with rapid economic development, the discharge of pollutants from industrial and agricultural production and the over-exploitation of lake water resources have become more and more common, resulting in the deterioration of lake water environments, water shortages, red tide outbreaks, and ecosystem degradation [2,3]. According to the Report on the State of the Environment in China in 2013, among the 26 key controlled lakes in China, 27.8% were eutrophic [4]. The eutrophication of lakes caused by the discharge of nutrients such as nitrogen and phosphorus can lead to the proliferation of phytoplankton and algae, reduced transparency, reduced dissolved oxygen, and the death of fish and other organisms, resulting in the loss of social and economic value along with ecological and environmental functions [5,6,7].

**Figure 1:**
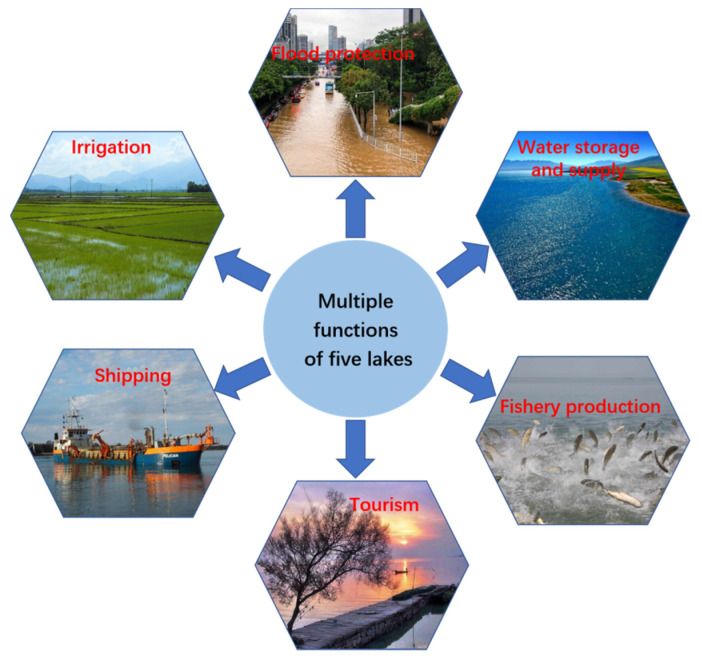
Multiple functions of the five lakes in the present study.

Jiangsu Province is one of the provinces with the largest number of freshwater lakes in China, containing more than 200 lakes of different sizes that occupy 6% of the total land area, the highest percentage in China [8]. Lakes are important sources of drinking water for the people of Jiangsu, with 21 lakes and reservoirs acting as water resources and accounting for 20.8% of all water sources in the province [8,9]. The five most famous lakes in Jiangsu are Taihu Lake (TL), Gehu Lake (GL), Gaobaoshaobo Lake (GSL), Hongze Lake (HZL), and Luoma Lake (LL), of which TL, HZL, and GSL are the third, fourth, and sixth largest lakes in China, respectively. GL is the second largest lake in southern Jiangsu Province, after TL; LL is not only the fourth largest freshwater lake in Jiangsu Province, but also serves as an important transit point for China’s south-to-north water transfer strategy [8,9]. These lakes have multiple important functions, such as flood protection, irrigation, water storage and supply, shipping, and fishery production (Figure 1). However, in the past decade, with rapid economic development and urbanization in Jiangsu, these lakes have experienced shrinkage of their free-water surfaces, increasing pollution loads, declining water qualities, and degradation of their natural ecosystems [10,11,12,13]. All of these changes represent increased safety risks to the drinking water sources in the region, which directly threaten the security of water resources and the health of all citizens. Meanwhile, Jiangsu is an important source of fish in China, and it had 76,000 hectares of aquaculture area in 2017 and produced the most freshwater fish of any province in China [14]. However, with the rapid development of lake aquaculture and increased fishery production load, there has been unrestricted discharge of associated industrial wastewater, domestic sewage, and farmland surface pollutants, and the fishery water environment has deteriorated as toxic substances accumulate, aquatic animal pests proliferate, and fishery pollution accidents occur more frequently, all of which affect the safety of aquatic products and increase the risks to human health [15,16]. Recently, a study conducted a comprehensive evaluation of the safety of typical lake drinking water sources in Jiangsu Province, and the results showed that the overall safety status of typical lake drinking water sources was relatively good, and the key indicators affecting the comprehensive safety of water sources were the various water quality parameters and ecological indicators [9]. Hence, understanding the current water quality, trends, and pollution statuses of these five lakes is essential for water conservation in the region, which is linked to economic development and the health of all citizens.

Over the past decades, the local government and environmental protection authorities have conducted environmental monitoring in the functional areas of TL, GL, GSL, HZL, and LL, including the diffusion zone, protected zone, net breeding zone, and inlet channel areas. However, there has been a lack of systematic water quality and ecological indicator monitoring analysis of all five lakes during different periods, which are not conducive to the protection of lake water and ecosystem because lakes are easily affected by the external environment due to their open and turbulent nature [17,18,19,20]. Furthermore, phytoplankton and zooplankton play important roles in aquatic ecosystems, and their community structures and functions are good indicators of aquatic ecological status [21,22]. Therefore, in order to serve as a reference for future regional industrial development, lake water environmental protection, and water quality control, this study utilized the eutrophication index, heavy metal concentrations, densities of zoo- and phytoplankton, and the benthic diversity index of the five lakes from 2013 to 2017 to study ecosystem health and dynamic trends of lakes in Jiangsu Province over five years. The results of this study are of great significance in the protection of drinking water resources, the safety of aquatic products, and the prevention and control of health risks.

## 2. Materials and Methods

### 2.1. Study Area

**Figure 2:**
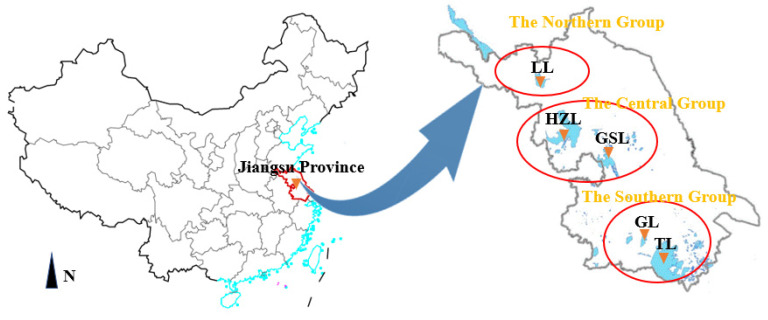
Locations of the five lakes in Jiangsu Province. Abbreviations stand for Taihu Lake (TL), Gehu Lake (GL), Gaobaoshaobo Lake (GSL), Hongze Lake (HZL), and Luoma Lake (LL).

The major lakes in Jiangsu Province are basically distributed on either side of the Beijing-Hangzhou Grand Canal. They are grouped in three main areas, to the south, in the center, and to the north of the Beijing-Hangzhou Grand Canal of China. The geographical locations of the five lakes are shown in Figure 2. The basic natural conditions of the five lakes, including total area, water volume, average water depth, average temperature, annual precipitation, and flood season, are shown in Table 1.

### 2.2. Sampling and Determination Methods

**Figure 3:**
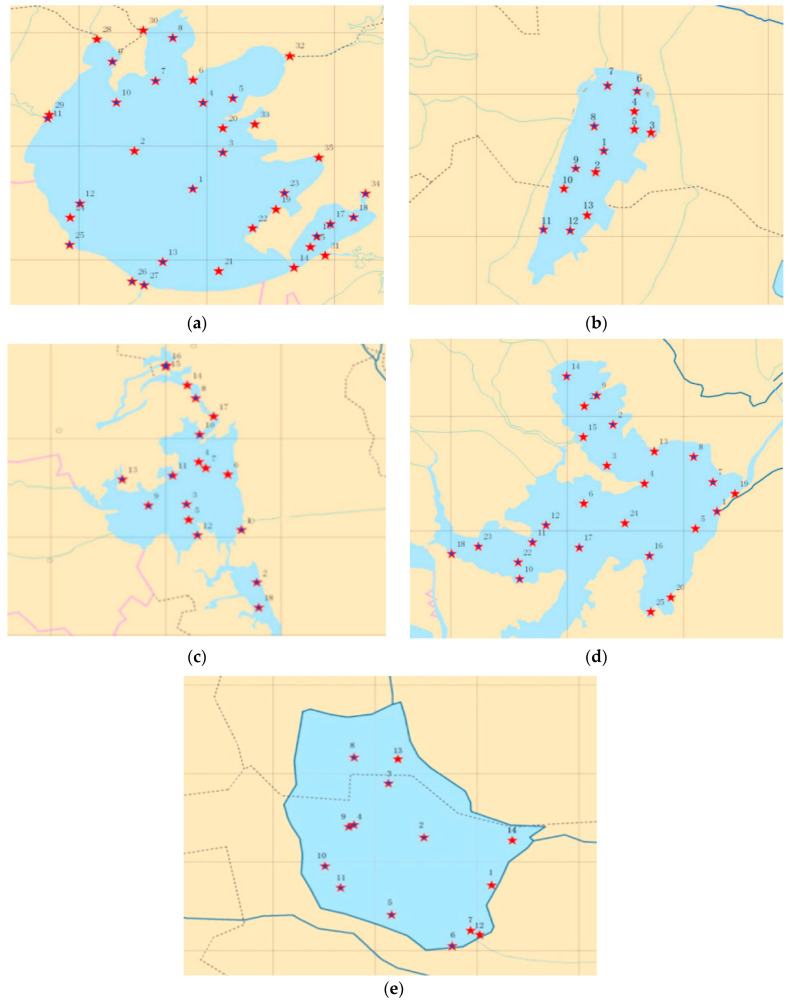
The locations of the sampling points in each lake. The abbreviations have the same meaning as Figure 1, (**a**) TL, (**b**) GL, (**c**) GSL, (**d**) HZL, and (**e**) LL. Red stars represent water quality detection sites and blue circles represent the sediment detection sites.

Water samples were collected during the normal-water period (April, May) and the high-water period (August, September) from 2013 to 2017. All samples were collected uniformly at the Jiangsu Fishery Ecological Environment Monitoring Station. The locations of the sampling points in each lake are shown in Figure 3. According to the area, specific functions, and spatial characteristics of each lake, 35 monitoring points were arranged in the river channel, open water area, protected area, and net enclosed aquaculture area of TL, 13 monitoring points were arranged in the open water area, entrance, and exit of the river and net enclosed aquaculture area of GL, 18 monitoring points were arranged in the open water area, net enclosed aquaculture area, protected area, and entrance of GSL, 25 monitoring points were set in the open water area, the entrance and exit of the lake, and the net enclosed aquaculture area of HZL, and 14 monitoring points were set in the open water area, the entrance and exit, and the aquaculture area of LL.

Glass bottles were used to collect water samples at each sampling point from 0.3 m below the lake surface. After sampling, 1 L of the water sample was immediately fixed with 15 mL of Lugol’s solution for subsequent phytoplankton quantitation. Then, 10-L water samples were filtered through a 64-micron filter to collect zooplankton and then fixed with 4% formalin for subsequent quantitation. After sampling, all samples were stored at 0–4 °C and analyzed within 12 h. A columnar sampler was used to collect sediment from the lake bottom at each sampling point. Sediment samples were put into a 50-mL centrifuge tube before cryopreservation, pretreatment, and analysis. Then, sediment samples were freeze-dried, and impurities, such as animal and plant residues and stones, were removed. Finally, all sediment samples were treated with a concentrated acid mixture (HNO_3_, HF, and HClO_4_) and then stored in amber glass vials before analysis [23].

The water sample pretreatment and analysis tests followed the relevant national standard methods [24]. Total nitrogen (TN) was determined using alkaline potassium persulfate digestion UV spectrophotometry (HJ636-2012). Total phosphorus (TP) was determined using ammonium molybdate spectrophotometry (GB11893-89), and chemical oxygen demand (COD) was determined using the acidic potassium permanganate method [25]. Concentrations of NH_3_-N were measured by Naismith spectrophotometry with medium-range (HI96715) (HANNA Instruments, Woonsocket, RI, USA). Petroleum was determined by ultraviolet spectrophotometry [26], and phytoplankton density and zooplankton density were analyzed according to the methods described by Jeong et al. [27].

The calculation of the Shannon-Wiener diversity index is as follows [28]:

(1) $$H\prime =-\sum \left(\frac{N_{i}}{N}\right)\cdot \ln\left(\frac{N_{i}}{N}\right)$$

where, H′ is Shannon Wiener’s diversity index, N_i_ is the number of individuals of a species collected at the sampling points in each lake, and N is the total number of individuals collected at the sampling points in each lake.

The Cu, Pb, and Cd concentrations in both water and sediment were determined by atomic fluorescence spectroscopy technique after acid digestion [29,30]. The concentrations of As and Hg were determined by arsenic molybdate-crystal UV spectrophotometry and cold atomic fluorescence after the samples dissolved in aqua regia [31,32,33].

### 2.3. Health Risk of Composite Assessment

TN, TP, NH_3_-N, COD, and oil content were selected as the five main parameters for evaluating water quality. The composite pollution index was calculated to evaluate the water quality of the five lakes.

The formula of the composite pollution index is as follows [24]:

(2) $$\mathrm{CPI}=\left[\frac{V_{\mathrm{TN}}}{S_{\mathrm{TN}}}+\frac{V_{\mathrm{TP}}}{S_{\mathrm{TP}}}+\frac{V_{{\mathrm{NH}}_{3}-N}}{S_{{\mathrm{NH}}_{3}-N}}+\frac{V_{\mathrm{COD}}}{S_{\mathrm{COD}}}+\frac{V_{\mathrm{Petrolsum}}}{S_{\mathrm{Petrolsum}}}\right]/5$$

where, CPI is composite pollution index, V (mg∙L^−1^) represents the values of various water quality monitoring parameters, and S (mg∙L^−1^) represents the water quality standards of the various water quality monitoring parameters.

The health risk assessment for freshwater lakes followed China’s Environmental Quality Standards for Surface Water (GB3838-88) (CEQSW) [34] and China’s Water Quality Standard for Drinking Water Sources (CJ 3020-93) (CWQSDWS) [35].

### 2.4. Data Analysis

Statistical analysis was performed using Microsoft Excel (Microsoft Corporation, Redmond, WA, USA) and the SPSS 26.0 software (SPSS Inc., Armonk, NY, USA). One-way analysis of variance (ANOVA) was performed to determine the statistically significant differences (*p* < 0.05). Tukey’s test was performed for pairwise comparisons. Pearson’s correlation coefficient was calculated, and a hypothesis test was performed to test the null hypothesis that the correlations among various water quality indicators were zero. All figures were constructed using Origin 9.0 (Origin Lab Corporation, Northampton, MA, USA). All data were reported as mean ± standard deviation.

## 3. Results

### 3.1. Concentrations of the Eutrophication Indicator and Petroleum

**Figure 4:**
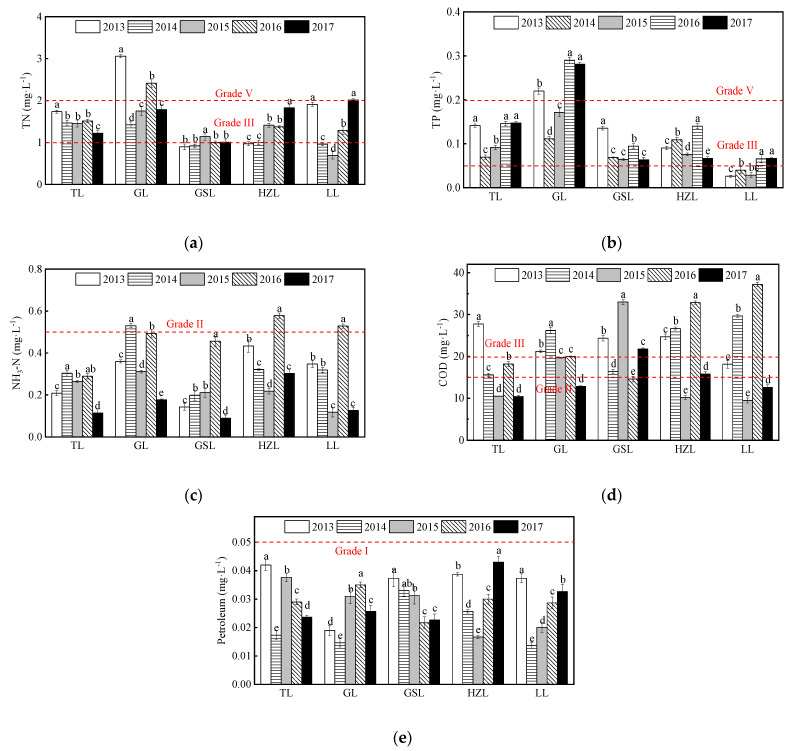
Annual average concentrations of eutrophication indicators and petroleum in the five lakes from 2013 to 2017. The abbreviations have the same meaning as Figure 1, (**a**) TN; (**b**) TP; (**c**) NH_3_-N; (**d**) COD; (**e**) petroleum. The red lines are the critical levels of indicator grades according to CEQSW. All data were reported as mean ± standard deviation. Different lowercase letters indicate significant differences among years (*p* < 0.05).

In 2013–2017, the TN concentrations in TL and GL exhibited fluctuating downward trends, while those of GSL and HZL exhibited fluctuating upward trends (Figure 4a). Among the five lakes, GL had the highest average TN concentration (2.09 mg∙L^−1^) over the five-year study period, which was classified as Grade V according to the CEQSW. GSL had the lowest average TN concentration (0.99 mg∙L^−1^) over the five years, which met the Grade III standard. For TP concentration during the study period (Figure 4b), the average concentration in GL (0.21 mg∙L^−1^) exceeded the Grade V standard according to the CEQSW; meanwhile, the lowest average value from LL (0.05 mg∙L^−1^) met the Grade III standard, although its TP concentrations increased with time. Moreover, although the TP concentrations of TL exhibited a fluctuating upward trend, the value never exceeded the critical level of Grade V of the CEQSW. Additionally, although the NH_3_-N concentrations in all five lakes were relatively high in 2016, they were all lower than 1.0 mg∙L^−1^ (critical level Grade IV) (Figure 4c). For the COD concentration, the average value from TL (16.49 mg∙L^−1^) was between Grade II and Grade III, while the average values from the other lakes (19.97–21.41 mg∙L^−1^) were close to the Grade V critical level according to the CEQSW (Figure 4d). On the other hand, lakes with aquaculture and shipping functions are usually polluted by petroleum due to their ship fishing and material transportation. The average petroleum concentrations in all five lakes in different years and over the five-year study period were below 0.05 mg∙L^−1^ (critical level Grade I), indicating that the five lakes had little petroleum pollution during these years (Figure 4e).

### 3.2. Density of the Phytoplankton and the Zooplankton

**Figure 5:**
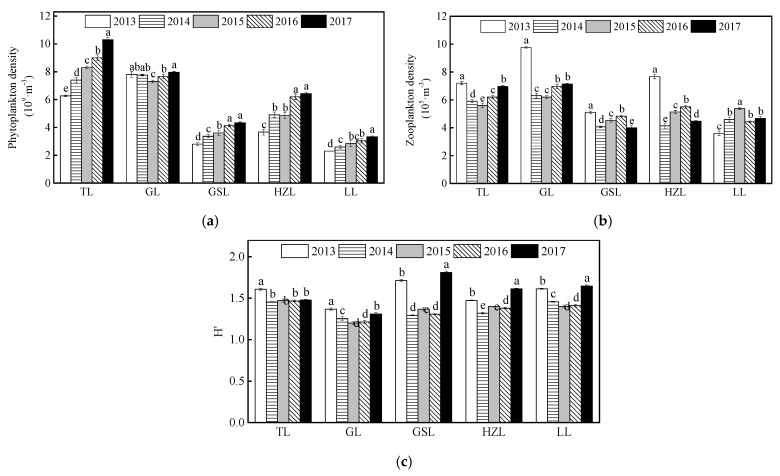
Plankton biodiversity in the five lakes from 2013 to 2017. The abbreviations have the same meaning as Figure 1, (**a**) phytoplankton density; (**b**) zooplankton density; (**c**) Shannon-Wiener diversity index. All data were reported as mean ± standard deviation. Different lowercase letters indicate significant differences among years (*p* < 0.05).

As shown in Figure 4, phytoplankton densities in TL, GSL, HZL, and LL increased from 2013 to 2017, while that in GL remained constant. The average phytoplankton densities in the five lakes followed the order: TL (8.25 × 10^9^∙m^−3^) > GL (7.70 × 10^9^∙m^−3^) > HZL (5.21 × 10^9^∙m^−3^) > GSL (3.65 × 10^9^∙m^−3^) > LL (2.82 × 10^9^∙m^−3^) (Figure 5a). The zooplankton densities decreased over time in GL, GSL, and HZL, while in TL and LL, the zooplankton exhibited high levels of fluctuation, either upward or downward. The average zooplankton densities in the five lakes followed the order: GL (7.27 × 10^5^∙m^−3^) > TL (6.37 × 10^5^∙m^−3^) > HZL (5.38 × 10^5^∙m^−3^) > LL (4.53 × 10^5^∙m^−3^) ≥ GSL (4.51 × 10^5^∙m^−3^) (Figure 5b). Additionally, diversity indexes of the zoobenthos in TL and GL exhibited negligible inter-year variability (Figure 5c).

### 3.3. Content of the Heavy Metals

**Figure 6:**
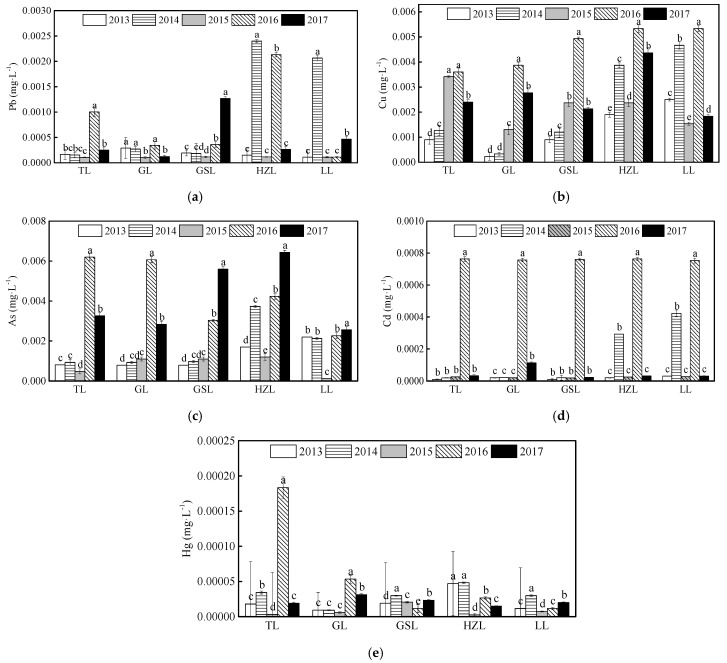
Annual average concentrations of the heavy metals in water of the five lakes from 2013 to 2017. The abbreviations have the same meaning as Figure 1, (**a**) Pb; (**b**) Cu; (**c**) As; (**d**) Cd; (**e**) Hg. All data were reported as mean ± standard deviation. Different lowercase letters indicate significant differences among years (*p* < 0.05).

Figure 5 illustrates how the concentrations of Pb, Cu, As, Cd, and Hg in the five lakes changed during 2013–2017. As observed, the concentrations of all heavy metals in this study were the significantly lower than the CWQSDWS, and the Pb, Cu, As, and Cd concentrations in the five lakes were significantly lower than the Grade I levels listed in the CEQSW (critical level Grade I is Pb ≤ 0.01 mg∙L^−1^, Cu ≤ 0.01 mg∙L^−1^, As ≤ 0.05 mg∙L^−1^, and Cd ≤ 0.001 mg∙L^−1^). Nevertheless, the Hg concentration in TL was 0.00018 mg∙L^−1^ in 2016, which was between Grade III (Hg ≤ 0.0001 mg∙L^−1^) and Grade IV (Hg ≤ 0.001 mg∙L^−1^), indicating it was a potential threat to aquatic animals and human beings (Figure 6e). Meanwhile, the Pb, Cu, and Cd concentrations increased significantly in HZL and LL in 2014, Cu, As, and Cd concentrations significantly increased in all five lakes in 2016, and As concentrations increased significantly in GSL and HZL in 2017. In summary, concentrations of different heavy metals in different lakes exhibited different trends.

**Figure 7:**
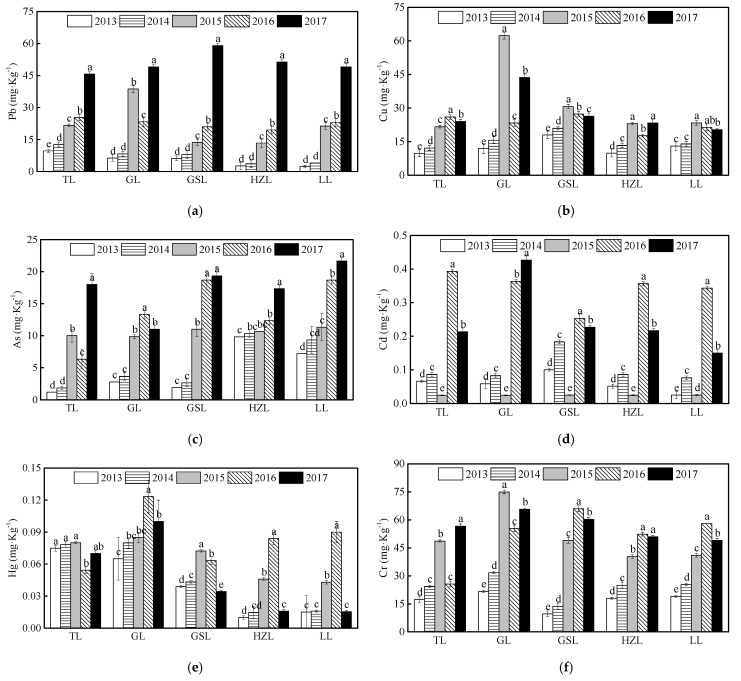
Annual average contents of the heavy metals in sediments of the five lakes from 2013 to 2017. The abbreviations have the same meaning as Figure 1, (**a**) Pb; (**b**) Cu; (**c**) As; (**d**) Cd; (**e**) Hg; (**f**) Cr. All data were reported as mean ± standard deviation. Different lowercase letters indicate significant differences among years (*p* < 0.05).

As shown in Figure 6, the contents of Pb and As in sediments from TL, GSL, HZL, and LL increased continuously from 2013 to 2017. Indeed, Pb contents in sediments from 2017 were four to five times of those from 2013 in all lakes (Figure 7a). Furthermore, accumulation of Cr and Cd was observed in the sediments of all five lakes from 2016 to 2017, and the concentration of Cu in of GL sediments markedly increased in 2015. Furthermore, Hg accumulation was universally observed in sediments of GL, GSL, HZL, and LL in 2016, but the Hg contents decreased drastically in 2017 in these lakes (Figure 7e).

### 3.4. Correlation Analysis of Various Water Quality Indexes and the Assessment of Health Risk

**Figure 8:**
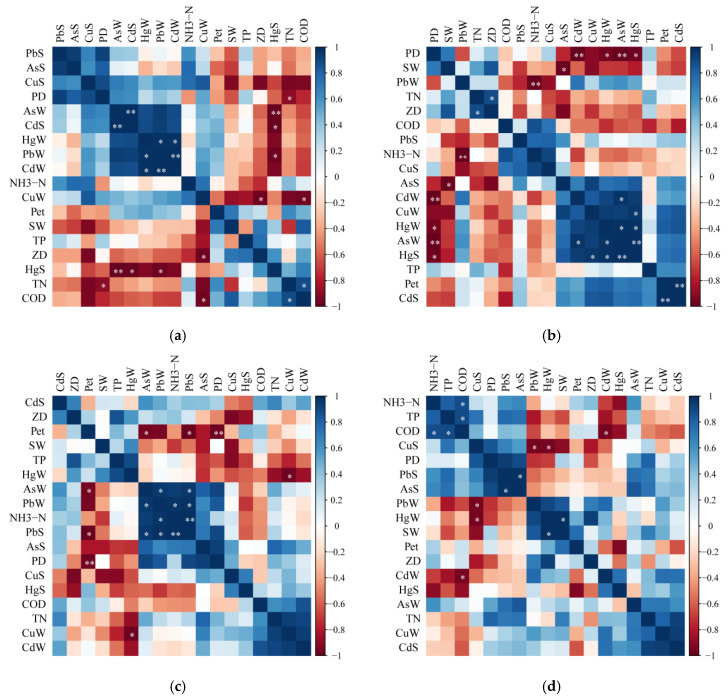

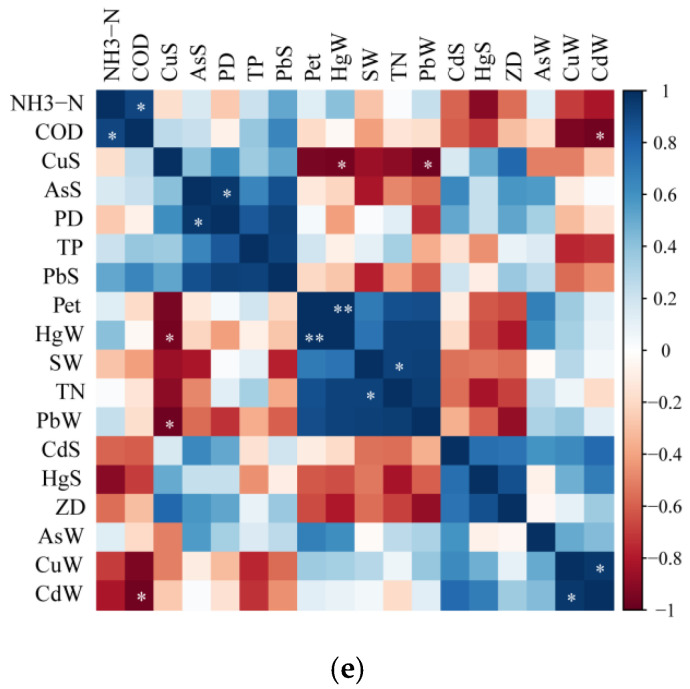
Correlations among various water quality indexes in the five lakes. PbW, CuW, AsW, CdW, and HgW represent the content of Pb, Cu, As, Cd, and Hg in water, and PbS, CuS, AsS, CdS, CrS, and HgS represent the contents of Pb, Cu, As, Cd and Hg in sediments. PD, ZD, and Pet represent phytoplankton density, zooplankton density, and petroleum concentration in water, respectively. ((**a**) TL; (**b**) GL; (**c**) GSL; (**d**) HZL; (**e**) LL; * significant correlation, *p* < 0.05; ** extremely significant correlation, *p* < 0.01).

The correlations among the various water quality indicators of the five lakes are shown in Figure 8; the blue and red colors between indicators represent positive and negative correlations, respectively. The deeper the color, the stronger the correlation (Figure 8). The results showed that there were common correlation characteristics among the five lakes. Firstly, positive correlations were found between the contents of heavy metals in the water body and in the sediment in all five lakes, suggesting that the change of heavy metal concentrations in water is consistent with that in sediment for all lakes. Secondly, positive correlations were found between the contents of TN and TP in the water bodies, both of which are important indicators of water eutrophication level. In addition, phytoplankton density was negatively correlated with the concentration of petroleum substances in all lakes. And significant positive correlations were also found between the phytoplankton densities and TN and TP contents in GL, GSL, HZL, and LL. These results indicated that petroleum pollution will lead to the reduction of phytoplankton in water, but the content of TN and TP in eutrophic water will stimulate the growth of phytoplankton, which is also the main reason for the outbreak of cyanobacteria in lakes [5,6,7]. Additionally, the zooplankton density and the Shannon-Wiener diversity index were negatively correlated with the concentrations of Pb, Cu, As, Cd, and Hg in both water and sediment from the five lakes, suggesting that heavy metal pollution in lakes will reduce the density and diversity of zooplankton in lakes. In TL, the contents of Hg were significantly positively correlated with contents of Pb and Cd (*p* < 0.05), and the positive correlation between Pb and Cd contents was highly significant (*p* < 0.01). Furthermore, in TL, a significant positive correlation was discovered not only between the zooplankton density and Cu, but also between the phytoplankton density and TN and COD (*p* < 0.05).

The composite pollution index is an important method for evaluating water quality [36]. According to formula (2), the CPI was calculated for all lakes (Table 2). The water quality index indicated that the five lakes were moderately polluted for most of the study period. The CPI of TL and GSL decreased over time, indicating that the water pollution had improved from moderate to light pollution status by 2017. Although the composite pollution levels of GL, HZL, and LL improved briefly, they returned to moderate pollution levels between 2016 and 2017.

## 4. Discussion

**Figure 9:**
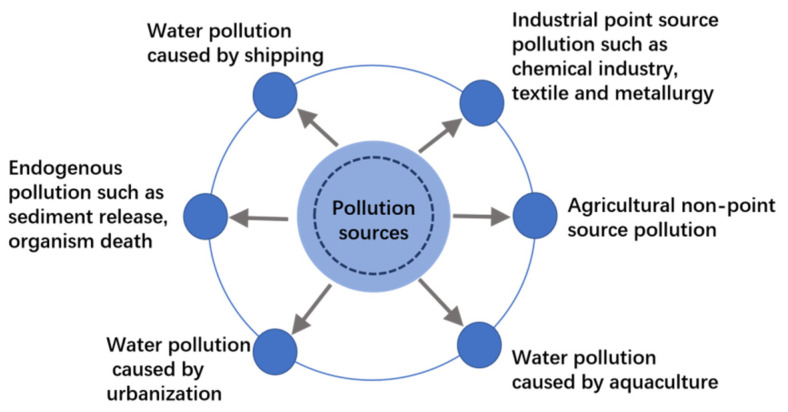
The major sources of pollution for the five lakes.

This study found that Jiangsu freshwater lakes had only minor pollution from petroleum substances during the 5-year monitoring period, which was mainly due to the limitations of the ship fishing and material transportation on the lakes. In addition, although the concentrations of TN and TP were positively correlated in all lakes, the removal of TP was the primary indicator of control and prevention measures in TL. GL had high average TN concentration over the five-year study period, but still met the Grade V critical level (CEQSW), while the average TP concentration (0.21 mg∙L^−1^) exceeded the Grade V critical level. This was due to the industrial point source pollution, such as the chemical textile, and metallurgy industries, the agricultural non-point source pollution, the aquaculture development, the urbanization of lake basin, and the endogenous pollution, such as sediment release and organism death (Figure 9). Furthermore, increases in lake aquaculture production in GL has increased TN and TP content, which has resulted in increased eutrophication [19,20]. The NH_3_-N concentration in lake and reservoir water sources is an important indicator that can be used to evaluate drinking water quality. During the five years of this study, the NH_3_-N concentrations in all lakes met both the Grade IV (1.0 mg∙L^−1^) in the CEQSW and the Grade II in the CWQSDWS. In addition, the NH_3_-N concentrations in TL, GL, HZL, GSL, and LL decreased in 2017 and all met the Grade I in the CWQSDWS. During the five years, the average COD concentration in TL (16.49 mg∙L^−1^) was classified as Grade III according to the CEQSW, much less than the other four lakes, which were close to Grade IV. The petroleum concentrations in all lakes were less than the value required for Grade I (0.05 mg∙L^−1^) in the CEQSW.

Phytoplankton are primary producers in water bodies, and their community structure and abundance directly determine the transfer of materials and energy along the food chain and the structure of aquatic ecosystems [37]. In 2013–2017, the phytoplankton densities increased in TL, GSL, HZL, and LL, while the phytoplankton densities remained stable in GL during these years. Among the lakes during the five-year study, the average phytoplankton density was highest in TL and lowest in LL. As we know, rapid phytoplankton reproduction, high water surface coverage, decreased water transparency, and reduced dissolved oxygen can lead to anoxic conditions, mortality in aquatic animals, and destruction of the aquatic ecosystem [38,39]. However, if the phytoplankton density is too low, it can result in a breakage of the food chain and affect the reproduction or biodiversity of zooplankton and create an imbalance in the ecosystem [40]. In these lake ecosystems, phytoplankton and zooplankton densities were related not only to the concentrations of water quality indicators, such as TN, TP, and COD, but also to local rainfall and lake water level [41,42,43]. Notably, although the concentration of petroleum was not high in any lake, phytoplankton density was highly sensitive to petroleum concentration, as indicated by the negative correlations between phytoplankton density and petroleum concentration in all lakes.

**Figure 10:**
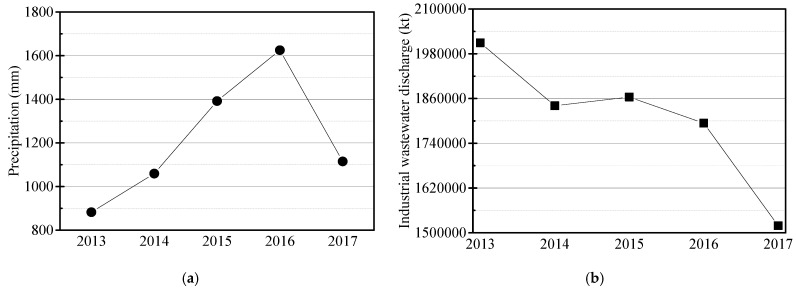
Annual average precipitation and annual discharge of industrial wastewater in Jiangsu Province in 2013–2017. (**a**) Annual average precipitation; (**b**) annual discharge of industrial wastewater.

The accumulation of heavy metals in aquatic environments is a great threat to the resident life. For example, heavy metals, such as Hg, Cd, and Cu, are characterized by long residency and accumulation times, bioaccumulation along food chains, and difficulty to remove; they not only poison aquatic organisms, but also threaten human health [44,45]. In this study, concentrations of different heavy metals in different lakes exhibited different trends. This can be attributed to two factors: (1) local industrial development and heavy metal emissions near the lakes; and/or (2) accumulation and release of heavy metals directly into the lakes. The zooplankton densities of all lakes were generally negatively correlated with the heavy metals Pb, Cu, As, Cd, and Hg in water. The concentrations of the heavy metals Pb, Cu, As, and Cd in all lakes were significantly lower than the value required to reach Grade I in the CEQSW between 2013 and 2017, indicating that the management and the control of heavy metals in those lakes had been comprehensively improved during this period. However, the concentration of Hg in TL reached a value as high as 0.00018 mg∙L^−1^ in 2016, which was in the Grade III-IV range of the CEQSW and posed potential risks to aquatic animals and humans [46]. At the same time, in 2016, the concentrations of Cu, As, and Cd in all lakes increased significantly. This was probably due to a combination of (1) the rapid development of agriculture and industry in Jiangsu, which resulted in a yearly increase in heavy metals discharged into the environment, and (2) the high rainfall in Jiangsu in 2016 (Figure 10a), which washed the heavy metals into the river systems and lakes. This not only resulted in increases in the concentrations of Cu, As, and Cd in all the lakes in this year, it also contributed to the accumulation of some heavy metals (e.g., Cr and Cd) in the sediment. Meanwhile, the contents of Pb and As in the sediments of TL, GSL, HZL, and LL showed a continuous increasing trend with time. This indicated that, although efforts had been made to control the discharge of industrial wastewater in Jiangsu Province to meet the Fisheries Water Quality Standard in China (GB 11607-1989) in 2013–2017 (Figure 8b), the pollution of heavy metals caused by release from sediments would continue to be a factor for the water quality and aquatic products in lakes for some time. In addition, the composite pollution index showed that the waters of the five lakes were moderately polluted for most of the monitoring period, indicating that the desired water quality of the lakes had not yet been achieved, and the protection of lake resources should not be relaxed for a long time in the future. In recent years, the Chinese government has amended the regulations on lake protection, which require strictly strengthening the lake management and protection, including water space control, resource protection, water pollution treatment, and water ecological environment improvement. Therefore, under the guidance of national policies and the joint efforts of Jiangsu provincial governments and environmental protection authorities at all levels, comprehensive efforts have been made to control water pollution in China from point-line-plane multidimensionality, and the water qualities of all freshwater lakes in Jiangsu Province have met China’s standards of both fishery water quality and drinking water quality.

## 5. Conclusions

However, these lakes are characterized by being open, regionally variable, and unstable. According to the present study from 2013 to 2017, although the concentrations of TN and TP were positively correlated in all lakes, the removal of TP was the primary indicator of control and prevention measures in TL. We suggest that the environmental protection department and the people living nearby should strengthen continuous supervision and management efforts to control the external input of nitrogen and phosphorus to avoid the recurrence of severe eutrophication in all lakes. We also found that the heavy metals in lakes released by sediments would be a main factor affecting water quality and aquatic products in the future. Thus, the concentration of heavy metals in the water of all five lakes water should be strictly monitored to ensure the heavy metals safety of water resources utilization when water in the lakes is used for fishery production, farmland irrigation, or drinking water supply, because prolonged release from the sediment to water could lead the enrichment of heavy metals in the human body through the food chain and endanger human health.

## Figures and Tables

**Table 1:** The basic natural conditions of the five lakes in Jiangsu Province.

Lake	Area (km^2^)	Water Volume (m^3^)	Average Water Depth (m)	Average Temperature (°C)	Annual Precipitation (mm)	Flood Season
TL	2338	4.4 × 10^10^	1.9	16–18	1110–1150	June–September
GL	146	0.215 × 10^10^	1.3	16	1126	June–September
GSL	960	(0.096–0.144) × 10^10^	1–1.5	14	1046	June–August
HZL	2069	3.12 × 10^10^	1.77	16.3	926	June–September
LL	296	0.27 × 10^10^	3.3	13.5	800	July–September

Note: The abbreviations are the same as Figure 1, the data are from the Report on the State of the Environment in China, and the URL link is https://english.mee.gov.cn/Resources/Reports/soe/ (accessed on 28 September 2019).

**Table 2:** Composite pollution index of the five lakes in 2013–2017.

	TL	GL	GSL	HZL	LL
2013	0.75	0.78	0.82	0.87	0.76
	Moderate	Moderate	Moderate	Moderate	Moderate
2014	0.73	0.64	0.70	0.72	0.73
	Moderate	Mild	Mild	Moderate	Moderate
2015	0.77	0.79	0.66	0.63	0.62
	Moderate	Moderate	Mild	Mild	Mild
2016	0.71	0.96	0.85	0.72	0.71
	Moderate	Moderate	Moderate	Moderate	Moderate
2017	0.69	0.75	0.68	0.77	0.76
	Mild	Moderate	Mild	Moderate	Moderate

Note: The abbreviations have the same meaning as Figure 1.

## Data Availability

All the data supporting this article were included in the main text.
